# Insect-Specific Virus Discovery: Significance for the Arbovirus Community

**DOI:** 10.3390/v7092851

**Published:** 2015-09-10

**Authors:** Bethany G. Bolling, Scott C. Weaver, Robert B. Tesh, Nikos Vasilakis

**Affiliations:** Institute for Human Infections and Immunity, Center for Tropical Diseases, and Department of Pathology, University of Texas Medical Branch, Galveston, TX 77555, USA; E-Mails: sweaver@utmb.edu (S.C.W.); rtesh@utmb.edu (R.B.T.); nivasila@utmb.edu (N.V.)

**Keywords:** insect-specific virus, arbovirus, evolution, vector competence, vaccine

## Abstract

Arthropod-borne viruses (arboviruses), especially those transmitted by mosquitoes, are a significant cause of morbidity and mortality in humans and animals worldwide. Recent discoveries indicate that mosquitoes are naturally infected with a wide range of other viruses, many within taxa occupied by arboviruses that are considered insect-specific. Over the past ten years there has been a dramatic increase in the literature describing novel insect-specific virus detection in mosquitoes, which has provided new insights about viral diversity and evolution, including that of arboviruses. It has also raised questions about what effects the mosquito virome has on arbovirus transmission. Additionally, the discovery of these new viruses has generated interest in their potential use as biological control agents as well as novel vaccine platforms. The arbovirus community will benefit from the growing database of knowledge concerning these newly described viral endosymbionts, as their impacts will likely be far reaching.

## 1. Introduction

Arthropod-borne viruses (arboviruses) are vertebrate-infectious viruses transmitted biologically (requiring replication in the vector) by mosquitoes, ticks, and other arthropod vectors. They are responsible for significant public health, social, and economic burden throughout the world, causing sporadic disease outbreaks and epidemics affecting human and animal populations. Some of the major contributors to this burden are mosquito-borne viruses like dengue (DEN), chikungunya (CHIK), yellow fever (YF), Japanese encephalitis (JE), West Nile (WN), and Rift Valley fever (RVF) viruses. Vaccines and antiviral drugs are lacking for most arboviral diseases and vector control efforts can be logistically and financially difficult to implement. Novel approaches are needed for vector control in order to reduce or prevent pathogen transmission.

Understanding microbial diversity in vector mosquitoes is important for control strategies. The growing interest and discovery of the diverse nature of the mosquito microbiome has given us new insights into the complex nature of vector-borne disease systems. *In vitro* and *in vivo* studies of newly described insect-specific viruses have revealed a novel group of viruses that are host-restricted to replication in invertebrate cells, in contrast to arboviruses, *sensu strictu*, which are able to replicate in both vertebrate and invertebrate cells. Phylogenetic analyses and experimental studies demonstrate that many of these insect-specific viruses isolated from mosquitoes are closely related to human pathogenic arboviruses, which raises questions about the potential role they may play in modulating arbovirus transmission. Ongoing studies characterizing these viruses indicate potential applications for biological control as well as novel vaccine strategies.

## 2. Detection of Insect-Specific Viruses

The term “insect-specific” refers to viruses that naturally infect mosquitoes and replicate in mosquito cells *in vitro*, but do not appear to replicate in vertebrate cells or to infect humans or other vertebrates. Insect-specific viruses were first described within the genus *Flavivirus*, family *Flaviviridae*. Cell fusing agent virus (CFAV), isolated 40 years ago, was the first insect-specific flavivirus characterized [1]. CFAV was isolated from culture fluid of an *Aedes aegypti* cell line, which produced massive syncytia when inoculated into a culture of *Aedes albopictus* cells, but it failed to replicate in three vertebrate cell lines (BHK-21, KB, and Vero) tested [1]. The complete nucleotide sequence for CFAV was determined approximately 15 years later and was found to be distantly related to other flaviviruses based on deduced amino acid sequences [2]. Sequence identities between CFAV and other flaviviruses were highest for the NS5 and NS3 genes, with 45% and 34% amino acid similarities, respectively. More recently, CFAV has been detected in field-caught *Aedes* and *Culex* spp. mosquitoes from Puerto Rico [3], Indonesia [4], Thailand [5], Mexico [6], and the United States (R. Tesh and N. Vasilakis, unpublished data). Phylogenetic analyses of the genus *Flavivirus* suggest that CFAV may represent a basal, possibly ancestral, lineage [2,7].

More than 25 years after the initial description of CFAV, a second virus, Kamiti River virus (KRV), was isolated from immature stages of *Aedes macintoshi* mosquitoes collected from flooded dambos in Kenya and was found to be related to CFAV [8,9]. Kamiti River virus also caused cytopathic effects (CPE) in *A. albopictus* (C6/36) cells, but not cell fusion, a hallmark of CFAV [8]. Similar to CFAV, KRV failed to replicate in vertebrate cells. Interestingly, sequences closely related to CFAV and KRV were found integrated into the genomes of laboratory-bred and field-caught *Aedes* spp. mosquitoes, generating questions about possible integration mechanisms involved and how these affect genetic diversity [10,11]. A third insect-specific flavivirus, Culex flavivirus (CxFV), was detected in 2007 in *Culex* spp. mosquitoes collected in Japan [12]. Isolates were made from *C. pipiens*, *C. tritaeniorhynchus*, and *C. quinquefasciatus* mosquito pools. Phylogenetic analysis of E protein amino acid sequences of CxFV and other flaviviruses revealed that CxFV clustered with CFAV and KRV in the insect-specific virus clade. Similar to previously described insect-specific viruses, CxFV did not infect vertebrate cells, but it also did not cause severe CPE in C6/36 cells, as seen with CFAV and KRV [12]. CxFV has since been isolated from numerous *Culex* species mosquitoes collected in many different parts of the world [13,14,15,16,17,18,19,20] and appears to be ubiquitous in nature.

**Figure 1:**
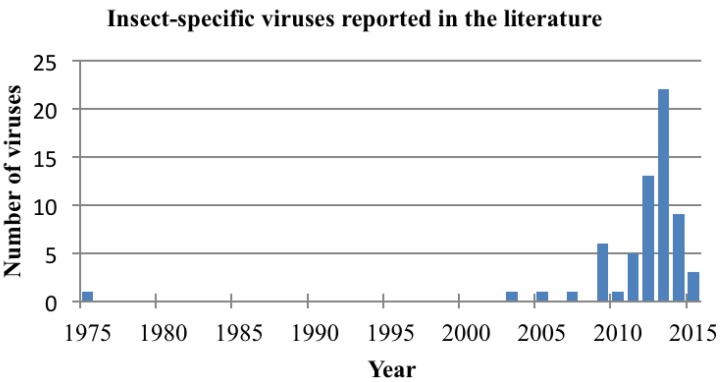
Timeline demonstrating dramatic increase in insect-specific virus discovery.

With advances in molecular tools for viral detection and also the growing interest into the mosquito microbiome, there has been a recent explosion in the detection and description of new insect-specific viruses (Figure 1). Most have been tentatively placed within the family *Flaviviridae* based on phylogenetic analyses but there have also been insect-specific viruses preliminarily classified in the families *Togaviridae*, *Rhabdoviridae*, *Bunyaviridae*, *Reoviridae*, *Mesoniviridae*, *Tymoviridae*, *Birnaviridae*, as well as other novel taxa (Table 1).

**Table 1:** Insect-specific viruses described in the literature.

Taxa	Virus	Isolation	Sequence	Reference
*Birnaviridae*	Culex Y virus Espirito Santo virus	Yes Yes	Full genome Full genome	[21] [22]
	Mosquito X virus	No	Full genome	[23]
*Bunyaviridae*	Cumuto virus	Yes	Full genome	[24]
	Gouléako	Yes	Full genome	[25]
	Herbert virus	Yes	Full genome	[26]
	Kibale virus	Yes	Full genome	[26]
	Phasi Charoen virus	Yes	Partial	[27]
	Tai virus	Yes	Full genome	[26]
*Flaviviridae*	Aedes cinereus flavivirus	No	Partial	[28]
	Aedes galloisi flavivirus	Yes	Partial	[29]
	Aedes flavivirus	Yes	Full genome	[4]
	Aedes vexans flavivirus	No	Partial	[28]
	Barkedji virus	Yes	Partial	[30]
	Calbertado virus	Yes	Partial	[16]
	Cell fusing agent virus	Yes	Full genome	[1,2]
	Chaoyang virus	Yes	Full genome	[31]
	Culex flavivirus	Yes	Full genome	[12]
	Culex theileri flavivirus	Yes	Full genome	[32]
	Czech Aedes vexans flavivirus	No	Partial	[28]
	Donggang virus	Yes	Full genome	Genbank accession #JQ086551 (2012)
	Hanko virus	Yes	Full genome	[33]
	Kamiti River virus	Yes	Full genome	[8,9]
**Taxa**	**Virus**	**Isolation**	**Sequence**	**Reference**
*Flaviviridae*	Lammi virus	Yes	Partial	[34]
	Mercadeo virus	Yes	Full genome	[35]
	Marisma mosquito virus	Yes	Partial	[36]
	Nakiwogo virus	Yes	Partial	[14]
	Nanay virus	Yes	Partial	[37]
	Nhumirim virus	Yes	Full genome	[38]
	Nounane virus	Yes	Full genome	[39]
	Ochlerotatus flavivirus	No	Partial	[28]
	Ochlerotatus caspius flavivirus	Yes	Full genome	[40]
	Palm Creek virus	Yes	Partial	[41]
	Quang Binh virus	Yes	Full genome	[42]
	Spanish Culex flavivirus	Yes	Partial	[36]
	Spanish Ochlerotatus flavivirus	Yes	Partial	[36]
	Yunan Culex flavivirus	Yes	Full genome	[43]
*Mesoniviridae*	Bontag Baru virus	Yes	Full genome	[44]
	Casuarina virus	Yes	Full genome	[45]
	Cavally virus	Yes	Full genome	[46]
	Dak Nong virus	Yes	Full genome	[47]
	Hana virus	Yes	Full genome	[48]
	Kamphaeng Phet virus	Yes	Full genome	[44]
	Méno virus	Yes	Full genome	[48]
	Moumo virus	Yes	Full genome	[48]
	Nam Dinh virus	Yes	Full genome	[49]
	Nsé virus	Yes	Full genome	[48]
Negeviruses	Dezidougou virus	Yes	Full genome	[50]
	Goutanap virus	Yes	Full genome	[51]
	Loreto virus	Yes	Full genome	[50]
	Negev virus	Yes	Full genome	[50]
	Ngewotan virus	Yes	Full genome	[50]
	Piura virus	Yes	Full genome	[50]
	Santana virus	Yes	Full genome	[50]
	Tanay virus	Yes	Full genome	[52]
	Wallerfield virus	Yes	Full genome	[23]
*Nodaviridae*	Mosinovirus	Yes	Full genome	[53]
*Reoviridae*	Aedes pseudoscutellaris reovirus	Yes	Full genome	[54]
	Cimodo virus	Yes	Full genome	[55]
	Fako virus	Yes	Full genome	[56]
*Rhabdoviridae*	Arboretum virus	Yes	Full genome	[57]
	Culex tritaeniorhynchus rhabdovirus	Yes	Full genome	[58]
	Moussa virus	Yes	Full genome	[59]
	Puerto Almendras virus	Yes	Full genome	[57]
*Togaviridae*	Eilat virus	Yes	Full genome	[60]
*Tymoviridae*	Culex tymovirus-like virus	Yes	Full genome	[61]

## 3. Viral Maintenance in Nature

Arbovirus transmission cycles have three essential components: the virus, the hematophagous arthropod vector, and the vertebrate host [62]. Arboviruses *sensu strictu* are maintained in nature by propagative biological transmission, where the virus replicates in two disparate systems: the invertebrate vector and the vertebrate host [63]. In contrast, insect-specific viruses lack the ability to replicate in vertebrate cells. To date, most insect-specific viruses have been isolated using C6/36 (*A. albopictus*) cells, with some studies attempting propagation in several different vertebrate cell lines in order to confirm this host-restriction characteristic [12,16,34]. Recent studies with an insect-specific alphavirus, Eilat virus (EILV), suggest that the basis for host-restriction is multigenic and viral replication is limited at multiple levels [64].

Data are lacking on the transmission dynamics of most insect-specific viruses in nature and the potential effects of these viral infections on mosquito life-history traits. Discovering how these viruses are maintained in nature and how they affect their mosquito hosts is important for understanding how they could potentially modulate arbovirus transmission. Several experimental studies have shown that insect-specific viruses can be transmitted vertically (from adult female to progeny) [65,66,67]; in addition, mosquito-specific viruses have been detected in immature stages and adult males from field collections [3,9,16,68] suggesting vertical maintenance in nature. Tissue tropisms of CxFV observed in *C. pipiens* [66] along with a recent study showing CxFV transmission from naturally infected males to naïve females [67] together indicate that venereal transmission may also play a role. These observations have been made with insect-specific flaviviruses and may not be representative of insect-specific viruses belonging to other virus families.

## 4. Viral Diversity and Evolution

**Figure 2:**
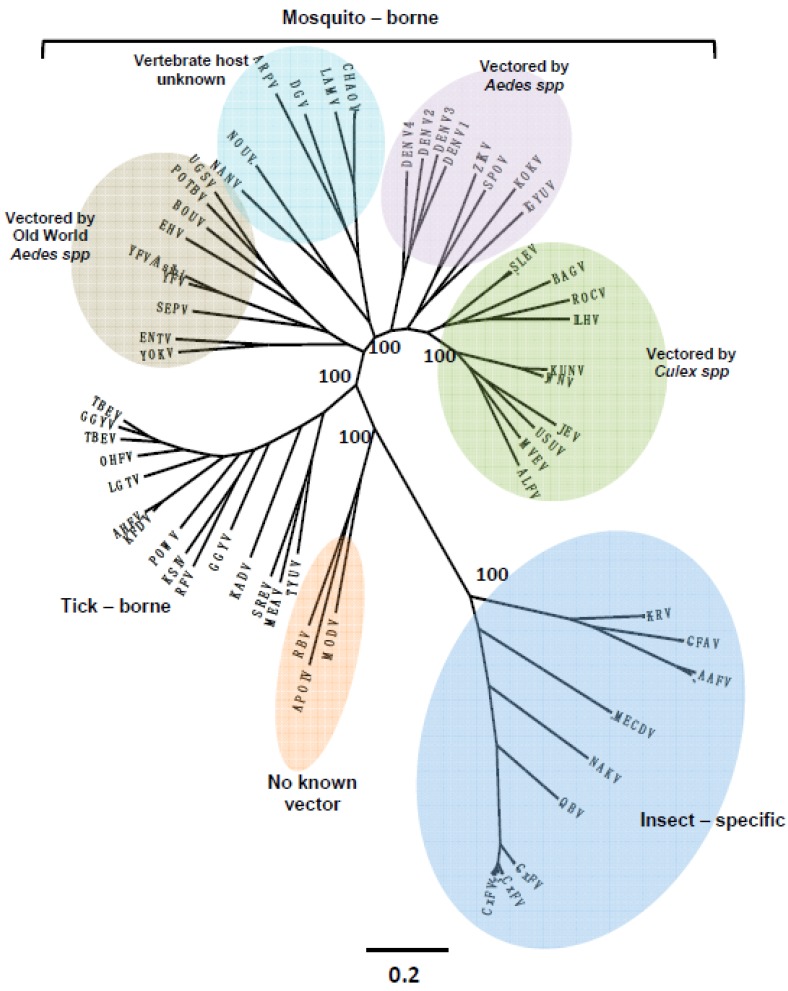
Flaviviruses: Maximum-likelihood analysis of select members of the genus *flavivirus*. Scale bars indicate amino acid substitutions/site. Branch labels indicate virus abbreviation. Additional virus isolate information is contained in a supplemental file.

**Figure 3:**
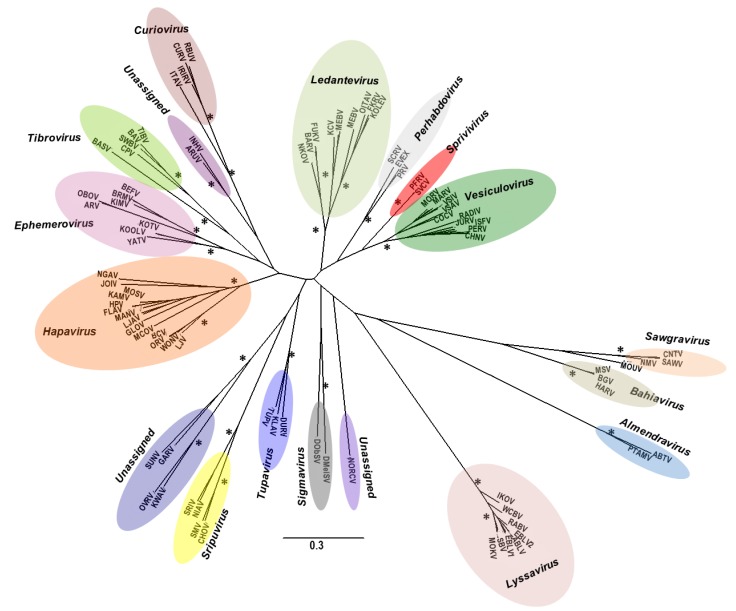
Rhabdovirus: Maximum-likelihood (ML) analysis of rhabdovirus L protein sequences. Horizontal branch lengths are drawn to a scale of amino acid substitutions/site, and all bootstrap support values ≥85% are indicated by an asterisk. Cytorhabdovirus, novirhabdovirus and nucleorhabdovirus outgroup sequences were excluded from the tree as they were too divergent to establish a reliable rooting. The tree is therefore rooted arbitrarily on one of two basal clades (genera Almendravirus and Bahiavirus) that comprise viruses isolated from mosquitoes. Branch labels indicate virus abbreviation. Additional virus isolate information is contained in a supplemental file. (Adapted from [73]).

**Figure 4:**
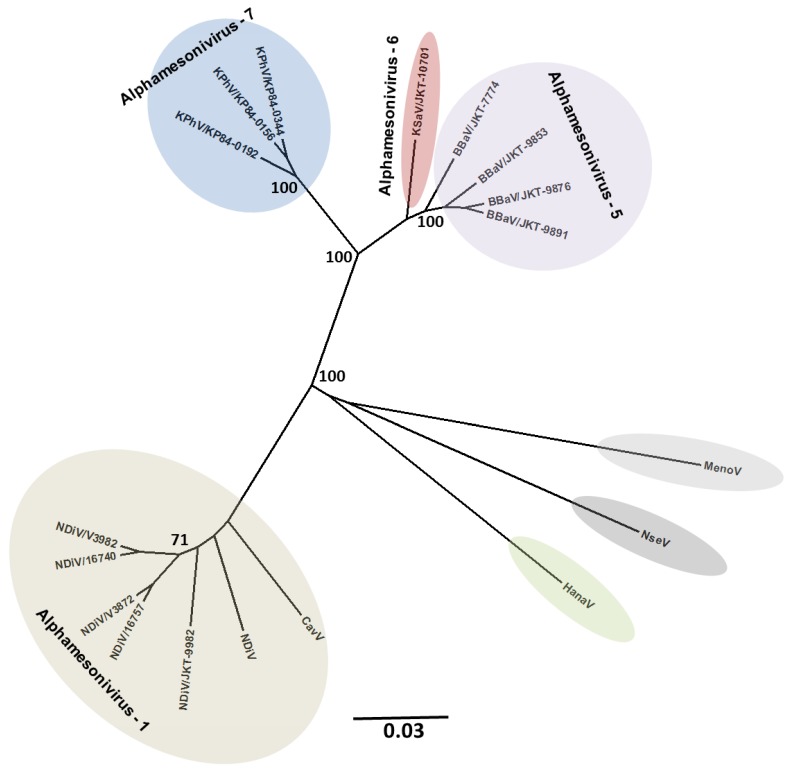
Mesoniviruses: Maximum-likelihood analysis of conserved protein domains of ORF1ab (3CLpro, RdRp, HEL1). Scale bars indicate amino acid substitutions/site. Branch labels indicate virus abbreviation/strain. Additional virus isolate information is contained in a supplemental file. (Adapted from [44]).

**Figure 5:**
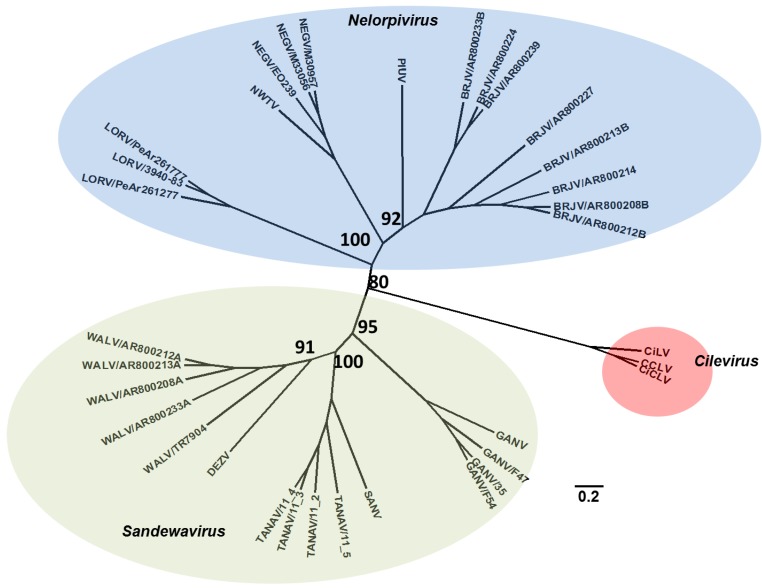
Negeviruses: Maximum likelihood analysis of select negeviruses. The region of the genome corresponds to the nt 4316–7309 (Negev E0239), which corresponds to the RNA-dependent RNA-polymerase of the genome. Branch labels indicate virus abbreviation/strain. Additional virus isolate information is contained in a supplemental file.

**Figure 6:**
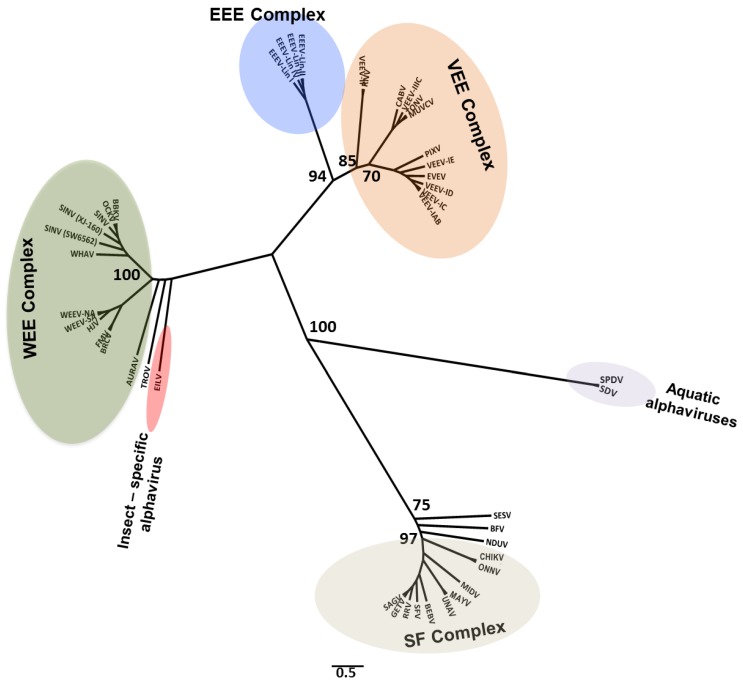
Togaviruses: Maximum likelihood analysis of select togaviruses. Phylogenetic tree based on nucleotide sequences of the alphavirus structural ORF. Branch labels indicate virus abbreviation/strain. Additional virus isolate information is contained in a supplemental file.

In addition to the classical arboviral pathogens such as dengue, yellow fever, chikungunya, Rift Valley fever and Japanese encephalitis viruses, mosquitoes are also infected with a wide variety of insect-specific viruses. This latter group is comprised of a growing number of largely RNA viruses, belonging to many different taxa including *Flaviviridae* (Figure 2), *Rhabdoviridae* (Figure 3), *Mesoniviridae* (Figure 4), negeviruses (Figure 5), and *Togaviridae* (Figure 6). Although insect-specific viruses do not infect vertebrates, they are nonetheless an important part of the mosquito microbiome. The recent development and wide availability of methods for full genome sequencing and metagenomics have revealed many novel insect-specific viruses [6,69,70,71,72]; yet little is known about their ecology or their effect on the mosquito host.

Recent phylogenetic studies [69,72,73] indicate that many of the RNA insect-associated viruses in the families *Bunyaviridae*, *Flaviviridae* and *Rhabdoviridae* are ancient with highly diverse lineages and that they probably evolved and diversified with their insect hosts [74,75]. The fact that many of these viral agents appear to be vertically transmitted is further evidence that they have been in contact with their insect hosts for a long period of time. Some appear to have become integrated with the genomes of their insect or arthropod hosts [10,76,77]. Others probably evolved from being insect-specific viruses to dual host viruses, capable of also infecting the animals or plants on which their insect hosts fed. Many arthropod-borne viruses of vertebrates and plants probably originated in this way [69,78]. Some viruses in this latter group may have completely adapted to animals or plants and lost the need for an insect host [62,69,78]. In view of their evolutionary history and plasticity, it seems likely that in the future other insect-specific will develop the ability to infect vertebrates or plants and become new emerging pathogens.

The concept that arboviruses originated in arthropods is not new. More than 50 years ago, Andrewes [79], Mattingly [80] and Maramorosch [78] all suggested that arboviruses originated from precursor viruses in arthropod vectors and that their introduction into vertebrates was a secondary or later development. Recent phylogenetic studies [69,72,73,74,75,76] demonstrate the high abundance and diversity of arthropod-associated RNA viruses, which supports the idea that arthropods may have played an important role in viral evolution, potentially serving as a hotspot where insect-specific viruses have evolved into dual-host viruses.

## 5. Effects on Vector Competence for Arboviruses

**Figure 7:**
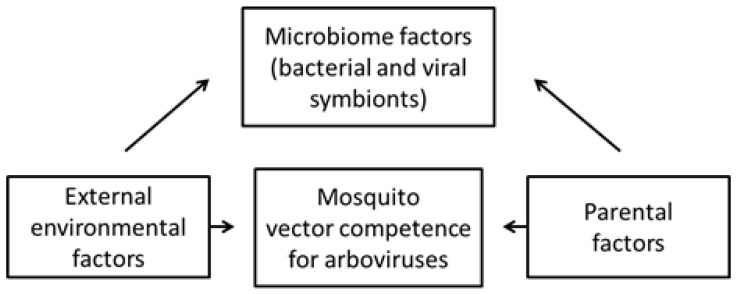
Mosquito vector competence for arboviruses can be affected by external and internal factors.

Much has been written about the replication of arboviruses in insects and insect cells, as well as descriptions of the innate and adaptive immune responses of insect cells to arbovirus infection [81,82]. In contrast, little is known about the response of insects or insect cells to infection with most of the newly discovered RNA insect-specific viruses. However, based on recent findings that bacterial symbionts of mosquitoes can alter the insects’ vector competence for certain arboviruses [83,84,85], some of the insect-specific viral symbionts of mosquitoes may have a similar effect (Figure 7), either as a result of superinfection exclusion [86,87,88] or alteration of the vector’s immune system [81,82].

Most of the reported studies of dual infection have examined the susceptibility of mosquitoes or mosquito cells that were infected with an insect-specific flavivirus, like CxFV, to superinfection with a flavivirus pathogen such as WNV or DENV. Results of these studies have been contradictory and inconclusive. For example, a retrospective study of *C. pipiens* mosquitoes collected in Chicago in 2006, found that WNV-positive mosquito pools had a four-fold increased likelihood of also containing CxFV compared to WNV-negative pools [89]. In contrast, Crockett and colleagues [90] found no evidence to support an association between WNV and CxFV prevalence rates in *C. quinquefasciatus* populations in the southeastern United States. *In vitro* and *in vivo* studies examining the potential interaction of WNV with insect-specific flaviviruses in mosquito cells and in mosquitoes have also produced conflicting results, highlighting the need for additional studies to clarify these interactions. Studies evaluating sequential infections of C6/36 (*A. albopictus*) cells, first with CxFV and followed by WNV 48 h later, resulted in significantly reduced WNV titers in co-infected cells, compared to controls [67]. A similar study looking at WNV replication kinetics in C6/36 cells co-infected with CxFV demonstrated slightly reduced WNV titers, but these differences were not significant [91]. Recent studies with a newly described insect-specific flavivirus, Palm Creek virus (PCV), isolated from *Coquillettidia xanthogaster* mosquitoes collected in Australia, showed suppression of WNV (Kunjin) and Murray Valley encephalitis virus replication in C6/36 cells that were persistently infected with PCV [41].

Nhumirim virus, another newly characterized insect-specific flavivirus, was found to reduce replication of WNV, Japanese encephalitis, and St. Louis encephalitis viruses in dually infected C6/36 cell cultures [92]. The *in vitro* experiments described above were all conducted using C6/36 cells, which, unlike live mosquitoes, do not have a functional antiviral RNAi response [93], so the biological relevance of these results is uncertain. In another series of experiments using a *C. tritaeniorhynchus* cell line chronically infected with CxFV, no evidence was found of superinfection exclusion upon infection with JEV or DENV [94]. The growth of DENV was similar in both CxFV-infected and non-infected *C. tritaeniorhynchus* cells; replication of JEV was also similar in the CxFV-infected and control cells for four days, although the cells superinfected with JEV eventually developed marked cytopathic effects (CPE) and the JEV titers declined. No CPE were observed in the JEV-infected control cells.

Only two published studies have looked at the effects of insect-specific virus infection on vector competence for arboviruses using live mosquitoes. The first study investigated vector competence of Florida *C. quinquefasciatus* mosquitoes for WNV, comparing females experimentally infected with CxFV to uninfected mosquitoes [91]. No significant effect on WNV replication were observed; however, a Honduran strain of *C. quinquefasciatus* inoculated simultaneously with CxFV and WNV demonstrated enhanced WNV transmission at 14 days post-infection [91]. Another study compared the vector competence for WNV of a Colorado strain of *C. pipiens* that was naturally infected with CxFV, to the vector competence of an Iowan strain of *C. pipiens* that was CxFV-free [67]. The dually infected mosquitoes showed significantly reduced WNV dissemination rates at seven days post-infection. The results of these studies are inconclusive and perhaps suggest that experiments investigating interactions between insect-specific viruses and arboviruses may vary depending on the mosquito and virus strains used. However, all of these experiments were done using insect-specific flaviviruses and there are many more insect-specific viruses belonging to other virus families and taxa that should be tested. Superinfection exclusion or homologous interference [86,87,88] is just one mechanism by which a viral symbiont might alter the vector competence of a mosquito vector for an arboviral pathogen.

## 6. Potential Novel Applications of Insect-Specific Viruses

### 6.1. Use of Insect-Specific Viruses as Biological Control Agents

Biological control measures involve the use of natural predators or pathogens to reduce mosquito abundance or vector competence. Examples of biological control used for mosquitoes include the larvivorous fish, *Gambusia affinis* [95], *Bacillus thuringiensis*, as a bacterial larvicide [96], and *Wolbachia*, maternally inherited bacterial endosymbionts present in many arthropod species which have been used to reduce some insect populations by way of cytoplasmic incompatibility [97]. Recent studies have shown the potential of certain *Wolbachia* strains to reduce the vector competence of mosquitoes by rendering them refractory to some human pathogens [98,99,100]. This finding represents one of the most promising new methods of biological control for mosquito-borne diseases. Other naturally occurring bacterial symbionts have also been shown to alter the ability of mosquitoes to become infected and to vector arboviral pathogens [83]. It is thought that the insect’s innate immune response is upregulated by the bacterial infection, and this immune activation results in protection against subsequent arboviral infection [84,85]. If a bacterial symbiont can alter the vector competence of a mosquito for arboviruses, then it seems likely that certain viral symbionts may have a similar effect [50]. Preliminary evidence suggests that some insect-specific viruses may alter the mosquito’s susceptibility to certain pathogenic arboviruses [41,67,91]. This interaction is not yet fully characterized and additional studies are needed to elucidate the dynamics of co-infection in mosquitoes. Since many insect-specific viruses are vertically transmitted, it may be possible to create a population of mosquitoes that is infected with an insect-specific virus, rendering them unable to transmit certain human pathogenic arboviruses. Further studies are warranted to investigate the potential for using insect-specific viruses as an innovative method for vector-borne disease prevention.

### 6.2. Use of Insect-Specific Viruses as Vaccine and Diagnostic Platforms

In addition to their potential use in disrupting arbovirus transmission, insect-specific viruses show potential as platforms for vaccine or diagnostic development. The insect-specific alphavirus, EILV [60], is defective for vertebrate cell infection at both the entry and RNA replication levels [64]. This inherent safety characteristic has been exploited to use recombinant DNA technology to generate EILV chimeras, where the structural polyprotein open reading frame is swapped with that of a vertebrate-pathogenic alphavirus to generate a chimera that is structurally indistinguishable from the latter virus. Although these chimeras enter vertebrate cells, they retain EILV’s restriction on RNA replication and thus are non-infectious for vertebrates. Chimeras between EILV and chikungunya virus (CHIKV) have been shown to serve as high quality antigens for enzyme-linked immunosorbent assays (ELISA), where they have many advantages including safety due to lack of vertebrate cell replication, which negates the need for inactivation, thereby preserving antigenic authenticity. They can therefore be produced cheaply and efficiently at biosafety level 1 containment in mosquito cells with titers generally exceeding 10^8^ infectious units/mL. An ELISA for CHIKV using an EILV/CHIKV chimera was shown superior to a commercial assay as well as to an ELISA using traditional antigens extracted from cell cultures (J. Erasmus and S. Weaver unpublished).

Eilat-based alphavirus chimeras have also been developed as vaccines for CHIKV and Venezuelan equine encephalitis (VEE). Single doses of 10^8^ infectious units have been shown to protect completely against lethal challenge with both CHIKV and VEEV in murine models, in some cases outperforming live-attenuated vaccines in immunogenicity. This remarkable efficacy appears to be mediated by the ability of the chimeric viruses to enter vertebrate cells by virtue of their structural proteins derived from CHIKV or VEEV, along with their packaging of subgenomic RNA that is translated into the structural proteins and presented on the surface of cells [101]. The ability of these chimeric genomes to encode an additional subgenomic promoter and efficiently express foreign genes [64], also suggests that they can be developed as highly safe yet rapidly immunogenic platforms for the development of vaccines against a wide range of infectious and even noninfectious diseases.

## 7. Conclusions

Vector-borne disease systems are ecologically complex, involving dynamic interactions between the pathogen, vertebrate host, and the vector, with the environment also playing an important role. Recent studies describing the presence of viral endosymbionts in mosquito populations give us new insight into the mosquito microbiome and how this “internal” environment may play an important role in regulating the transmission of important pathogens. Our growing understanding about the diverse nature of these insect-specific viruses has thus far generated more questions than answers. Further studies are warranted to continue to elucidate the role that these viral endosymbionts play in nature and also investigate how we can take advantage of their unique properties for arbovirus control efforts.

## Supplementary Files

Supplementary File 1
